# 

**Published:** 1898-03

**Authors:** 


### CONTENTS.

ORIGINAL COMMUNICATIONS—

The Diagnosis and Treatment of Appendicitis. By Louis Frank, M.D., Louisville, Kentucky ... 1

National vs. State Quarantine. By W. D. Travis, M.D., Covington, Ga. ... 10

The Experiences and Reminiscences of Forty Years. By G. G. Roy, M.D., Atlanta, Georgia ... 14

Otomyasthenia—Muscle-Deafness. By Thomas F. Rumbold, M.D., St. Louis, Mo. ... 18

Some Remarks on Fistula in Ano. By William S. Goldsmith, M.D., Atlanta, Ga. ... 23

SOCIETY REPORTS—

The Quarantine Convention of the South Atlantic and Gulf States, at Mobile, Alabama ... 27

CORRESPONDENCE—

Compensation for Services ... 35

Painless Vaccination ... 36

Information Wanted about Coca ... 37

EDITORIAL—

The Mobile Quarantine Convention ... 39

Law to Prevent Ophthalmia Neonatorum ... 41

The Plea of Insanity ... 43

Meeting of the Southern Section of the American Laryngological, Rhinological and Otological Society, in Atlanta, Ga., March 28—Preliminary Program ... 45

MEDICAL ITEMS ... 49

BOOK REVIEWS ... 53

SELECTIONS AND ABSTRACTS ... 60

MEDICAL ITEMS—Continued ... x, xi, xii, xiii, xiv, xvi, xvii

ADVERTISERS' INDEX TO ADVERTISEMENTS ... xviii

CLASSIFIED INDEX TO ADVERTISEMENTS ... iii

